# Exploring the Cardiovascular Potential of Artichoke—A Comprehensive Review

**DOI:** 10.3390/biology14040397

**Published:** 2025-04-10

**Authors:** Henrique Silva, Avina Mahendra Daia

**Affiliations:** 1 Research Institute for Medicines (iMed.ULisboa), Faculdade de Farmácia, Universidade de Lisboa, Av. Prof. Gama Pinto, 1649-003 Lisbon, Portugal; 2 Department of Pharmacy, Pharmacology and Health Technologies, Faculdade de Farmácia, Universidade de Lisboa, Av. Prof. Gama Pinto, 1649-003 Lisbon, Portugal; 3 Biophysics and Biomedical Engineering Institute (IBEB), Faculdade de Ciências, Universidade de Lisboa, Campo Grande, 1749-016 Lisbon, Portugal

**Keywords:** *Cynara cardunculus*, cardiovascular health, supplementation, hypertension, endothelial function, renin–angiotensin–aldosterone system

## Abstract

Cardiovascular disease remains a leading cause of morbidity and mortality worldwide, requiring both pharmacological and lifestyle-based preventive strategies. Artichoke (*Cynara cardunculus* L. var. scolymus) has gained attention for its health benefits, including choleretic and lipid-lowering activities. However, its cardiovascular effects remain underdiscussed. This paper provides a critical review of the current literature on the cardiovascular effects of artichoke, with a focus on its underlying mechanisms of action and clinical efficacy. Experimental studies assessing artichoke’s effects on endothelial function, vascular smooth muscle relaxation, and modulation of the renin–angiotensin–aldosterone axis were assessed. Additionally, clinical studies, systematic reviews, and meta-analyses investigating its antihypertensive effects were reviewed. Artichoke and its bioactive components, particularly flavonoids and caffeoylquinic acids, enhance endothelial-dependent and -independent vasorelaxation and inhibit angiotensin-converting enzyme activity. Although clinical studies indicate improvements in flow-mediated dilation, they report only modest reductions in blood pressure, with high variability in formulations, dosages, and patient populations. While artichoke supplementation may support blood pressure regulation and endothelial health, current evidence suggests it should be considered an adjunct rather than a replacement for conventional antihypertensive therapy. Standardized formulations and well-controlled clinical studies will be required to clarify its therapeutic role.

## Simple Summary

Cardiovascular disease is one of the leading causes of illness and death worldwide. While pharmacological strategies play a key role in managing cardiovascular health, lifestyle changes, including a healthy diet, are also essential. Artichoke has been used for centuries in cooking and traditional medicine, and recent research suggests it may have benefits for cardiovascular health. This review explores artichoke’s potential to improve cardiovascular health, specifically its antihypertensive effects. Experimental studies show that several bioactive artichoke compounds induce vasorelaxation and suppress the renin–angiotensin–aldosterone axis. Although clinical studies indicate improvements in flow-mediated dilation, they report only modest reductions in blood pressure, with high variability in formulations, dosages, and patient populations. Although artichoke supplementation may improve overall cardiovascular health, it should not be considered a substitute for conventional antihypertensive drugs, but rather as part of a healthy lifestyle. More research is needed to confirm its effectiveness.

## 1. Introduction

Cardiovascular diseases are highly prevalent worldwide, particularly in developed countries, and are associated with significant mortality, morbidity, and substantial healthcare costs [1,2,3]. As the prevalence of cardiovascular diseases is expected to rise in the coming decades, the implementation of preventive strategies becomes imperative to slow disease progression and mitigate associated complications. Effective management of cardiovascular diseases requires a multifaceted approach, encompassing the timely identification and control of risk factors as well as the optimization of existing pharmacotherapeutic strategies. In this regard, there is an ongoing need for the development of novel and safer drugs, along with the refinement of drug administration protocols. Beyond pharmacological interventions, the adoption of and long-term adherence to a healthy lifestyle, including regular physical activity, a balanced diet, and stress management, play a pivotal role in disease prevention [4]. The Mediterranean diet is frequently recommended by healthcare professionals as a valuable complement to pharmacological therapy [5]. It emphasizes the consumption of a wide range of nutrient-rich foods, particularly vegetables and fruits, many with cardioprotective properties [6].

**Figure 1:**
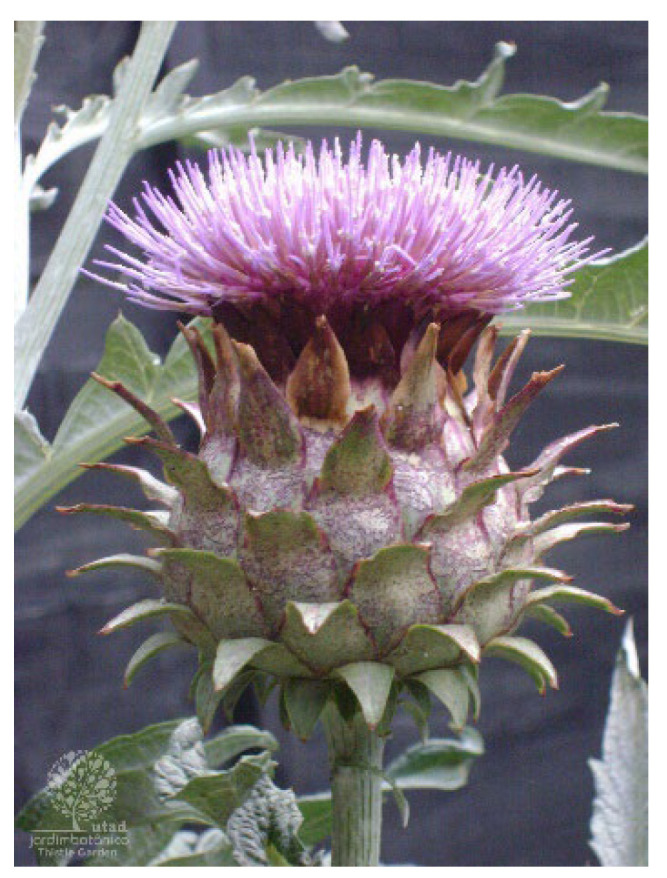
*Cynara cardunculus* L. var. scolymus (Thistle garden, from Jardim Botânico UTAD, Flora Digital de Portugal).

Among the many foods in the Mediterranean diet, the artichoke is known for its nutritional and therapeutic benefits, but it is still not widely recognized [7]. This vegetable has been consumed for centuries, with historical texts referencing its culinary use dating back to the Classical Period [8,9,10]. *Cynara* is a small genus within the *Asteraceae* family comprising eight species and four subspecies, including the thistle or cardoon (*Cynara cardunculus* L.) [11,12,13]. The thistle has three botanical varieties: the artichoke (Figure 1), also known as the globe artichoke (*Cynara cardunculus* L. var. scolymus); the cultivated or leafy thistle/cardoon (*Cynara cardunculus* L. var. altilis); and the wild thistle/cardoon (*Cynara cardunculus* L. var. sylvestris). The globe artichoke, formerly known as *Cynara scolymus* L., is a perennial herbaceous crop native to the Mediterranean region, where it has been cultivated for thousands of years before spreading worldwide. The plant’s scientific name originates from the Greek word *skolymos*, meaning “pointed stake”, in reference to its spines, while *kynara* may derive from the name of an Aegean island where it was once grown [9]. The artichoke’s life cycle can exceed 10 years, although intensive farming practices often reduce it to 2–4 years [11]. Its stem is very short, and its leaves can reach lengths of 50–200 cm. The inflorescence, also known as the capitulum or head, comprises a long peduncle that can reach up to 180 cm in length, a receptacle where the flowers are inserted, and external bracts (i.e., fleshy leaves). Each plant produces a primary head along with 4–20 secondary and tertiary heads. The bracts serve to protect the flower itself. At the base of the flower bud lies the “heart”, which, along with the base of each bract, forms the edible portion of the artichoke [14]. Above the heart lies the “choke”, a mass of hair-like structures that will eventually develop into the plant’s flowers. 

Artichokes are considered a culinary delicacy, and in certain countries they are a national dish [15,16,17]. Their appreciation dates back centuries, with historical accounts suggesting that even Caravaggio (1571–1610), the famed baroque painter, had a particular fondness for this vegetable—so much so that he allegedly got into a heated dispute over how it should be prepared [9,18]. Leaves, external bracts, and stems, which comprise approximately 60–85% of the plant’s biomass, are unsuitable for human consumption and are therefore generally considered waste in the industrial processing of artichoke [19,20,21,22]. Nevertheless, efforts are being made to maximize the utilization of discarded parts across various industries. Leaves, external bracts, and stems can serve as sources of food additives and nutraceuticals [19,20] or as raw materials in green chemistry industries, including paper pulp production, biofuels, and plant-based dyes [13,23]. Additionally, artichoke by-products have been explored as ingredients for bread production [24,25], contributing to the development of functional foods with enhanced nutritional value. Moreover, artichokes can also be used as minimally processed vegetables [26], offering a convenient and nutritious option for ready-to-eat consumption. Artichoke seed oil is used in the production of soap, shampoo, alkyd resin, shoe polish, and biodiesel [27,28]. In recent years, growing interest has also emerged in the enzyme content of artichokes, as several products are known to contain enzymes with proteolytic activity, which are utilized in cheese production [29].

In healthcare, the artichoke is well known for its digestive properties. Specifically, they are used to treat gallbladder disorders, indigestion, and nausea [30]. Additionally, they have been shown to lower plasma cholesterol levels, particularly low-density lipoproteins (LDL) [31], although this effect may depend on individual genetic factors, especially those related to lipoprotein composition and transport [32]. Owing to this effect, artichoke leaf extracts have been proposed as a nutraceutical option for patients with statin intolerance, offering a potential alternative for LDL management in selected cases [33]. Artichoke also exhibits hepatoprotective effects, partly attributed to the presence of inulin, and the European Medicines Agency officially recognizes its use for this purpose. Clinical trials suggest that artichoke extracts may have therapeutic potential in treating several conditions, including metabolic dysfunction-associated steatotic liver disease (formerly known as non-alcoholic fatty liver disease, NAFLD) [34], hypercholesterolemia [35], and metabolic syndrome [36,37]. These health-promoting effects have been the focus of several recent reviews [7,38,39,40]. However, one lesser-known benefit of artichokes is their ability to improve cardiovascular health. A growing body of research highlights several beneficial effects, including improvement in flow-mediated dilation (FMD) and reduction in blood pressure. Supporting the potential relevance of these findings at the population level, an ecological study conducted in several municipalities of the Valencian Community (Spain) found that higher local production of artichoke—used as a proxy for consumption—was associated with lower mortality from cardiovascular diseases [41]. Although ecological studies cannot establish causality, this observation aligns with the hypothesis that artichoke consumption may contribute to cardiovascular prevention.

The blood pressure-lowering effects of the artichoke have been documented in three recent systematic reviews and meta-analyses [42,43,44]. However, the high heterogeneity among the included studies, encompassing differences in population characteristics, study design, and intervention protocols, poses challenges to the interpretation of their findings. These inconsistencies limit the reliability of the reported effects, thereby warranting a more critical assessment of the evidence. In addition, the specific mechanisms underlying the antihypertensive effects of the artichoke remain insufficiently explored, and other potential cardiovascular benefits have not been fully addressed. Therefore, this paper aims not only to critically assess the existing evidence but also to provide a comprehensive review of the cardiovascular therapeutic potential of the artichoke, with a particular focus on elucidating the mechanisms responsible for these effects and identifying directions for future research.

## 2. Composition of Artichoke Extracts

The composition of artichoke-derived products varies significantly depending on the part of the plant used, as well as factors such as geographic location, plant age, and harvesting, processing, and storage methods. For a more in-depth discussion of the processing variables that influence composition, refer to Colombo et al. (2024) [45]. The most bioactive-rich parts include the bracts, stems, residual leaves, roots, and seeds. Table 1 summarizes the main bioactive compounds identified for each part of the artichoke.

The bracts are among the most studied parts of the plant and are primarily composed of fibers (cellulose, hemicellulose, lignin, and inulin), proteins, and polyphenols, particularly phenolic acids such as caffeoylquinic acids (CQAs) and dicaffeoylquinic acids (diCQAs), with chlorogenic acid (5-CQA) and cynarin (1,3-diCQA) being two of the most representative compounds. These phenolics, along with flavones like luteolin and apigenin [46,47], have demonstrated antioxidant and vasodilatory properties, which may contribute to the cardioprotective effects of the artichoke. Additionally, bracts contain sesquiterpene lactones (e.g., cynaropicrin), which are responsible for the characteristic bitter taste and have been linked to digestive and hepatoprotective properties [48,49]. The concentration of these bioactive compounds varies based on plant maturity and the position of the bracts, with the inner bracts, closer to the “heart” of the artichoke, being the richest in polyphenols [50]. Stems and residual leaves are notable for their high fiber content, including both insoluble (cellulose, hemicellulose, and lignin) and soluble fibers (inulin, pectin, gums, and β-glucans), which may provide gut health and metabolism [43,44]. They also contain flavanols, namely proanthocyanidins, as well as low amounts of sesquiterpene lactones [49]. The presence of peroxidases in the stems suggests additional antioxidant activity [45]. Residual leaves are particularly rich in proteolytic enzymes (cardosins and cyprosins), which have applications in the food industry [29,51], as well as flavonoids and phenolic acids (CQAs, diCQAs) [52]. Although stalks, roots, and seeds are less studied, they also contain valuable bioactive compounds. Stalks contain fibers (cellulose, hemicellulose, and lignin), with their caliber determining the proportion of these compounds [53]. Roots are particularly rich in inulin, with concentrations varying according to cultivar and growing conditions [54]. Seeds contain proteins, polyunsaturated and monounsaturated fatty acids (e.g., linoleic and linolenic acids), phenolic acids (CQAs and diCQAs), terpenoids (e.g., tocopherols), phytosterols (e.g., β-sitosterol, campesterol, and 5-stigmasterol), and minerals (calcium, potassium, magnesium, manganese, iron, sodium and zinc), making them a potential source for nutraceutical and industrial applications [55,56,57].

The main products made from artichoke include ethanolic and aqueous extracts from leaves and essential oils from seeds, all being suitable for human consumption. Aqueous extracts retain higher levels of hydrophilic compounds such as flavonoids and phenolic acids, whereas ethanolic extracts extract higher levels of lipophilic compounds such as terpenoids. Artichoke seed oil is particularly rich in unsaturated fatty acids [58,59]. It is important to note that most studies evaluating the biological activity of artichoke compounds are based on purified extracts or in vitro assays, which do not always reflect the biochemical profile of artichoke as consumed in typical diets. Artichoke is often subjected to thermal processing (e.g., boiling, steaming) or incorporated into complex formulations, both of which may alter the chemical composition and bioactivity of its components. It has been reported that common practices such as steaming and boiling increase the concentrations of CQAs, while frying decreases the concentration of flavonoids [60]. Additionally, the interaction of these compounds with the food matrix or other dietary constituents may influence their bioavailability. These considerations highlight the need for further studies assessing the stability and pharmacokinetics of bioactive artichoke compounds under realistic preparation and consumption conditions.

Beyond the effects of processing, gastrointestinal digestion and metabolism also influence the systemic availability of artichoke-derived compounds. In vitro digestion models suggest that most polyphenols retain their antioxidant activity post-digestion, although certain diCQAs may be affected [61]. Studies using Caco-2 intestinal cells have shown that flavonoids like luteolin can cross the epithelial barrier, while CQAs may require enzymatic cleavage to become absorbable [62]. Chlorogenic acid appears resistant to gastric degradation and may be absorbed in the stomach, though microbial metabolism in the colon also contributes to its absorption [63,64]. Colonic fermentation combined with Caco-2 permeation models indicates that flavonoids have greater intestinal bioavailability than chlorogenic acid or cynaropicrin [65]. These in vitro findings are supported by human pharmacokinetic data showing that artichoke compounds are excreted not as parent molecules, but as conjugated metabolites (e.g., sulfates and glucuronides of caffeic and ferulic acids) [66]. Taken together, these observations underscore the importance of considering both compound transformation and metabolite bioactivity when evaluating systemic effects.

## 3. Cardiovascular Effects of the Artichoke

### 3.1. Endothelium-Protecting Effects In Vitro

The endothelial vascular layer plays several important roles in vascular homeostasis. Under physiological conditions the endothelium is responsible for the secretion of important vasodilator mediators, the expression of proteins that prevent platelet adhesion, and mediating leukocyte migration, among others [67]. Conversely, under pathophysiological conditions, the endothelium secretes vasoconstrictor mediators and exposes platelet adhesive proteins. Both artichoke and several of its bioactive compounds are known to display endothelium-protective activity via different mechanisms of action. Some studies demonstrate that artichoke is able to increase the secretion of NO. In human umbilical vein endothelial cells (HUVECs), artichoke extracts have been demonstrated to increase the release of nitric oxide (NO) via potentiation of the mRNA expression of endothelial NO synthase (eNOS) [68]. In aortic endothelial cells, an artichoke extract increased NO release by maintaining an intracellular reduced state, increasing the intracellular levels of cofactors such as tetrahydrobiopterin (BH_4_), which prevents eNOS uncoupling [69]. Besides NO, artichoke also stimulates the endothelial secretion of prostacyclin (PGI_2_) [70]. Other studies describe that artichokes prevent the degradation of NO by reactive oxygen species (ROS) via their antioxidant activity. In HUVECs, an artichoke extract prevented the generation of ROS by lipopolysaccharide (LPS) and oxidized LDL in a concentration-dependent way [71]. A second study showed the same, with an ethanolic extract of artichoke reducing oxidized LDL-induced ROS generation in a more potent way than an aqueous extract [69]. This endothelium-protecting activity seems to be attributed to the presence of flavonoids, namely luteolin, as well as phenolic acids such as CQAs [68]. Cynarin (1,3-di-O-caffeoylquinic acid), a major bioactive compound, protects endothelial EA.hy926 cells (i.e., a cell line derived from HUVECs) by decreasing the inflammation evoked by LPS, specifically by decreasing the expression of vascular cell adhesion molecule 1 (VCAM-1) and proinflammatory mediators such as monocyte chemoattractant protein 1 (MCP-1), tumor necrosis factor α (TNF-α), and interleukin 1β. Also, cynarin inhibits the activation of p38 and nuclear factor kappa B (NF-κB) pathways by inducing the negative regulator mitogen-activated protein kinase phosphatase 3 (MKP-3) [72].

### 3.2. Endothelium-Protecting Effects Ex Vivo and In Vivo

**Figure 2:**
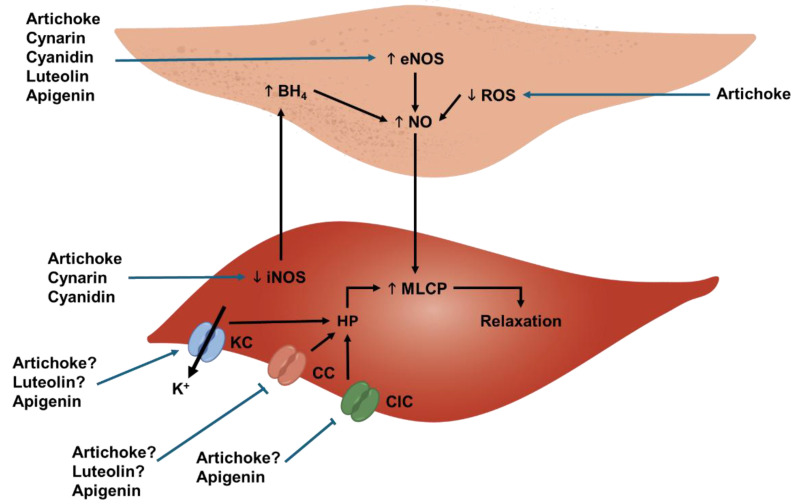
Scheme of the probable vasorelaxation mechanisms of artichoke and its most relevant bioactive compounds. An endothelial cell is represented at the top and a vascular smooth muscle cell at the bottom (BH_4_—tetrabiopterin; CC—calcium channel; ClC—calcium-mediated chloride channel; eNOS—endothelial nitric oxide synthase; HP—hyperpolarization; iNOS—inducible nitric oxide synthase; KC—potassium channel; MLCP—myosin light-chain phosphatase; ROS—reactive oxygen species).

Studies carried out ex vivo have demonstrated that artichoke extracts and several of their bioactive compounds display vasorelaxant effects. Currently, these effects seem to be attributed to the potentiation of the endothelial release of NO and to the direct relaxation of the vascular smooth muscle. The known mechanisms underlying the vasorelaxant effects of artichoke and its most relevant bioactive compounds are presented in Figure 2. An artichoke leaf extract was found to potentiate the vasorelaxant effects of acetylcholine in endothelium-intact rat aorta [68], suggesting the existence of a mechanism that increases NO secretion [73]. Several bioactive artichoke compounds are thought to display this mechanism of action, namely cynarin, cyanidin, luteolin, and apigenin. Cynarin is known to relax rat aorta [74] whereas cyanidin-3-rutinoside, a cyanidin glucoside, relaxes rat aorta and mesenteric arteries [75]. Also, when administered to anesthetized rats, cyanidin-3-rutinoside significantly reduced blood pressure [75]. Similarly, luteolin might also induce vasorelaxation, since it was found to directly upregulate eNOS, either as an aglycone or as the 7-glucoside derivative (cynaroside) [34,76]. In addition to increasing endothelial NO secretion, bioactive artichoke compounds may also inhibit their inactivation by ROS. Furthermore, artichoke may also indirectly improve endothelial-mediated vasorelaxation by downregulating inducible NOS (iNOS) in vascular smooth muscle (VSM) cells, which may be attributed to cynarin or cyanidin [73]. During vascular inflammation, iNOS expression increases, leading to higher consumption of BH_4_, thereby reducing its availability for eNOS and limiting the vasorelaxant potential of the endothelium [77,78]. As such, by downregulating iNOS, artichoke may lessen the impact of inflammation and improve overall vascular function.

In endothelium-denuded arteries, an artichoke leaf extract was also found to evoke a vasorelaxant action, suggesting a direct effect in the VSM [70]. This extract also improved vasodilation in aged rats. Some bioactive artichoke compounds are known to act in VSM cells, namely luteolin and apigenin. Luteolin evokes endothelium-independent vasorelaxation in the rat thoracic aorta [79,80,81] and uterine arteries [82] by inhibiting calcium channels and activating potassium channels in the VSM, leading to cell hyperpolarization and relaxation. Apigenin also relaxes rat aorta [83,84] and intra-renal [85], mesenteric [86], and pial arteries [87] by mechanisms involving the modulation of transient receptor potential channels (TRP) channels, potassium channels, and chloride or calcium channels [85].

### 3.3. Modulation of the Renin–Angiotensin–Aldosterone Axis

The renin–angiotensin–aldosterone (RAA) endocrine axis plays a central role in long-term blood pressure regulation by modulating vascular tone and blood volume [88]. The increase in renal sympathetic activity or the decrease in renal perfusion pressure leads to the renal secretion of renin, an enzyme that converts angiotensinogen into angiotensin I. Angiotensin-converting enzyme (ACE), present in the renal and pulmonary circulations, then transforms angiotensin I into angiotensin II, a potent vasoconstrictor that also stimulates the release of aldosterone from the adrenal cortex. Aldosterone promotes sodium and water retention in the kidneys, increasing blood volume and blood pressure, and also contributes to cardiac remodeling. Chronic activation of the RAA axis is a key driver of hypertension and other cardiovascular diseases, making it a common target for pharmacological intervention.

Recent in vitro studies have demonstrated that an artichoke leaf extract and certain bioactive compounds inhibit ACE activity [89], an effect attributed to luteolin and apigenin [90]. Additionally, enzymatic hydrolysates derived from artichoke, when applied to dairy products, have been shown to generate bioactive peptides with ACE-inhibitory properties [91,92]. These findings suggest that artichoke-derived compounds could contribute to blood pressure regulation through mechanisms similar to ACE inhibitors. Artichoke may exert ACE-inhibitory activity and influence other endocrine regulators of cardiovascular function. Notably, such inhibition has been associated with reduced circulating leptin levels [93], a hormone linked to endothelial dysfunction and obesity-related hypertension [94]. However, the direct impact of artichoke on leptin signaling remains unexplored. Future research should investigate whether artichoke supplementation can modulate RAA activity and leptin levels in vivo, particularly in hypertensive and metabolically compromised populations.

### 3.4. Improvement in Flow-Mediated Dilation

Flow-mediated dilation (FMD) is a widely used measure of endothelial function, reflecting the ability of blood vessels to release NO and induce vasodilation. This process can be assessed in vivo using transient arterial occlusion followed by cuff release, a method known as post-occlusive reactive hyperemia [95]. Impaired FMD is associated with an increased risk of cardiovascular disease, making it a valuable marker for vascular health.

Table 2 summarizes the most relevant characteristics of the clinical studies that assessed the effects of artichoke on FMD.In a study by Lupatelli et al. (2004) [96], hyperlipidemic subjects consumed artichoke juice for six weeks. Compared to controls, the artichoke group exhibited significant improvements in FMD and reductions in circulating levels of vascular adhesion molecules, including VCAM-1 and intercellular adhesion molecule 1 (ICAM-1). Similarly, Castellino et al. (2019) administered a nutraceutical supplement containing artichoke extract to individuals with metabolic syndrome over six months and reported a significant improvement in FMD [97]. Terzo et al. (2023) used the same supplement in individuals with pre-obesity, also for six months, and likewise observed a significant increase in FMD [98]. Notably, both studies additionally reported a significant reduction in carotid intima-media thickness, suggesting that this formulation may have consistent vascular protective effects across different at-risk populations. Additional evidence comes from a study by Maurotti et al. (2024) [99], in which subjects with non-alcoholic liver steatosis received a combination of artichoke and bergamot for three months. The intervention resulted in a significant increase in the reactive hyperemia index. Likewise, Fogacci et al. (2022) [100] reported an increase in endothelial reactivity after administering a complex supplement containing artichoke, among other compounds, for one month. However, these findings should be interpreted with caution. The studies by Maurotti et al. (2024) [99] and Fogacci et al. (2024) [100] involved combination supplements, making it difficult to isolate the effects of artichoke from those of other bioactive ingredients. Future studies should focus on evaluating the isolated effects of artichoke using standardized formulations and controlled study designs.

### 3.5. Possible Effect of Reduction in Body Weight and Insulin Resistance

Besides directly influencing physiological determinants of blood pressure, artichoke may also exert an indirect antihypertensive effect through weight reduction, a mechanism that has been underexplored in the literature. It is well established that weight loss lowers blood pressure by reducing peripheral vascular resistance and cardiac output. In fact, several hypertension management guidelines recommend weight reduction as a primary lifestyle intervention before pharmacological treatment [101,102]. However, the extent to which artichoke contributes to weight loss remains unclear, as studies evaluating this effect have yielded inconsistent findings. Ardalani et al. (2018) [76], Panahi et al. (2018) [34], and Ferro et al. (2020) [103] reported a significant reduction in body mass index (BMI) or body weight in groups treated with artichoke compared to control groups. However, other studies found no significant differences between intervention and control groups [35,104,105], suggesting that this effect may depend on factors such as intervention duration, participant metabolic profile, and the specific formulation of artichoke extract used. Further research is needed to determine whether artichoke plays a significant role in weight management and how this might translate to blood pressure regulation.

### 3.6. Antihypertensive Activity

The mechanisms by which artichoke reduces blood pressure are not yet fully understood. Current evidence suggests that artichoke and its bioactive compounds exert antihypertensive effects by enhancing endothelial secretion of vasodilators (NO and PGI_2_) and directly relaxing VSM, thereby reducing peripheral vascular resistance [106]. Additionally, studies indicate that certain artichoke-derived compounds may inhibit ACE, potentially contributing to blood pressure reduction by modulating the RAA axis [88]. Beyond its direct vascular effects, artichoke could also influence blood pressure indirectly by reducing body weight and insulin resistance.

The antihypertensive effects of artichoke in human subjects have been analyzed in several systematic reviews and meta-analyses [42,43,44], but findings have been inconsistent. Moradi et al. (2021) reported that artichoke supplementation significantly lowered blood pressure, but primarily in hypertensive and hyperlipidemic patients [42]. In contrast, Phimarn et al. (2024), after reviewing a larger dataset, concluded that artichoke supplementation reduced systolic blood pressure, but had no clear effect on diastolic blood pressure [44]. These discrepancies may be explained by methodological differences, as some meta-analyses included studies where artichoke was administered in combination with other herbal supplements [103,107] or as an adjunct to existing antihypertensive medications [35,76,108]. Meanwhile, a meta-analysis by Amini et al. (2022), which excluded studies featuring co-administered supplements, also found a significant reduction in blood pressure with artichoke supplementation [43].

One major challenge in interpreting these findings is the substantial variability among the included studies. Table 3 summarizes the most relevant characteristics of the clinical studies included in these meta-analyses, along with other relevant studies published thereafter. The studies evaluated the effects of artichoke in highly diverse populations, including individuals with isolated hypercholesterolemia [35], overweight prehypertensive subjects [108], and patients with metabolic conditions such as non-alcoholic fatty liver disease [34] and non-alcoholic steatohepatitis [105], as well as those with diagnosed hypertension [76,104]. While all of these conditions contribute to cardiovascular risk, their pathophysiological mechanisms differ, which may explain the variability in the clinical efficacy of artichoke across studies [109,110]. Furthermore, there was considerable variation in the composition, formulation, dosage, and treatment duration of artichoke products across studies, complicating the interpretation of results. Treatment durations were two months [34,35,76,105,107], three months [103,104], or six months [108]. Additionally, in several studies, artichoke was co-administered with other plant extracts or supplements [35,97,107], making it difficult to determine whether the observed effects were attributable to artichoke itself. Finally, among the nine clinical trials reviewed, only three reported a statistically significant reduction in blood pressure [76,104,105], and the magnitude of this effect was rather modest (~1–4 mmHg). On its own, such a modest reduction may be insufficient to significantly reduce cardiovascular risk, particularly in patients with moderate-to-severe hypertension. Moreover, the reported magnitude of blood pressure reduction may have been influenced by differences in measurement procedures. Thus, while artichoke may complement dietary and lifestyle modifications for blood pressure control, it is unlikely to serve as a replacement for antihypertensive medications with well-established efficacy and safety profiles. Future research should aim to standardize formulations, define optimal dosages, and investigate potential interactions between artichoke supplementation and other therapeutic strategies to clarify its role in hypertension management.

### 3.7. Safety Profile of Artichokes

Artichoke products, particularly in dietary amounts, are generally considered safe. However, clinical data on long-term supplementation remain limited [111]. Common adverse reactions associated with artichoke supplementation include diarrhea, abdominal spasms, nausea, heartburn, and flatulence [111,112]. These effects may be related to its influence on bile secretion and digestion. Allergic reactions have been reported, particularly in occupational exposure settings, where individuals handling fresh or dried artichoke plants have experienced eczema-like skin reactions. However, the prevalence of such reactions remains unclear. The possibility of cross-reactivity between artichoke and other plants of the *Asteraceae* family has not been reported. While no cases of oral allergic reactions to ingested artichoke have been documented, caution is advised in sensitive individuals. Due to its bile-stimulating (choleretic) effect, artichoke products should not be used by individuals with bile duct obstruction, including those with gallstones, as they could exacerbate symptoms or lead to complications [112]. Although artichoke has been consumed safely for centuries as a food, standardized supplements and extracts require further investigation to fully characterize their long-term safety and potential interactions with medications.

## 4. Conclusions

Artichoke exhibits cardiovascular therapeutic potential, supported by its vasorelaxant effects, ACE-inhibitory activity, and clinical evidence of improved flow-mediated dilation (FMD). However, there is a lack of well-designed, large-scale randomized controlled trials evaluating its isolated effects on blood pressure. Many studies used heterogeneous populations and variable artichoke formulations, making it difficult to draw definitive conclusions. The available evidence suggests that artichoke supplementation leads to only modest reductions in blood pressure, typically in the range of 1–4 mmHg. While this may be beneficial for individuals at risk of cardiovascular disease, these effects are likely insufficient as a standalone intervention for patients with hypertension. Given its modest effects, artichoke should currently be considered a complementary strategy rather than a replacement for conventional antihypertensive therapies. It may have a role in combination with other lifestyle interventions to promote cardiovascular health. Future studies should prioritize well-controlled trials using standardized artichoke formulations and identifying patient populations that may benefit the most from supplementation, while mechanistic research should further explore its role in RAA axis modulation and endothelial function.

## Figures and Tables

**Table 1:** Main constituents of the different parts of artichoke.

Part	Compounds
Bracts	Fibers (cellulose, hemicellulose, lignin, and inulin) Proteins Phenolic acids—CQAs (e.g., chlorogenic acid) and diCQAs (e.g., cynarin) Flavones (e.g., luteolin, apigenin) Terpenoids—sesquiterpene lactones (e.g., cynaropicrin)
Stems	Fibers (cellulose, hemicellulose, lignin, inulin, pectin, gums, β-glucans) Peroxidase enzymes Proanthocyanidins Terpenoids (sesquiterpene lactones)
Residual leaves	Fibers (cellulose, hemicellulose, lignin, inulin, pectin, gums, β-glucans) Proteolytic enzymes (cardosins, cyprosins) Flavanols (proanthocyanidins) Terpenoids—sesquiterpene lactones Phenolic acids (CQAs, diCQAs)
Stalks	Complex carbohydrates (cellulose, hemicellulose, and lignin)
Roots	Fibers (e.g., inulin)
Seeds	Proteins Polyunsaturated and monounsaturated fatty acids (e.g., linoleic and linolenic acids) Phenolic acids (CQAs and diCQAs) Terpenoids (e.g., tocopherols) Phytosterols (e.g., β-sitosterol, campesterol, 5-stigmasterol) Minerals (calcium, potassium, magnesium, manganese, iron, sodium and zinc)

**Table 2:** Main results of the studies describing the effect of artichoke supplementation on flow-mediated dilation (FMD; CG—control group, TG—treatment group, y.o.—years old).

Authors (Year)	Population	Artichoke Product	Intervention	Main Results
Lupatelli et al. (2004) [96]	28 hyperlipidemic subjects (TG: N = 18, 53 y.o.; CG: N = 10, 55 y.o.)	Artichoke leaf juice	20 mL/day for 6 weeks	Significant increase in FMD when compared with the control group
Castellino et al. (2019) [97]	100 subjects with metabolic syndrome (TG: N = 50, 63 y.o.; CG: N = 50, 63 y.o.)	Nutraceutical containing extracts of two artichoke species (Altilix^®^, Belpasso, Italy)	150 mg/day for 6 months	Significant increase in FMD when compared with the control group
Terzo et al. (2023) [98]	50 subjects with pre-obesity (TG: N = 28; CG: N = 22; undisclosed ages)	Nutraceutical containing extracts of two artichoke species (Altilix^®^, Belpasso (CT), Italy)	150 mg/day for 6 months	Significant increase in FMD when compared with the control group
Maurotti et al. (2024) [99]	32 subjects with non-alcoholic liver steatosis (TG: N = 16, 51 y.o.; CG: N = 16, 52 y.o.)	Supplement (300 mg) containing artichoke extract and bergamot polyphenol fraction (Bergacyn^®^, Bianco, Italy)	1 capsule/day for 3 months	Significant increase in the reactive hyperemia index when compared with the control group
Fogacci et al. (2024) [100]	90 subjects with hypercholesterolemia (TG: N = 45, 46.2 y.o.; CG: N = 45, 47 y.o.)	Nutraceutical containing a bergamot extract (1000 mg), two artichoke extracts (120 mg), coenzyme Q10 (5 mg) and zinc (5 mg) (Eufortyn^®^ Colesterolo Plus, Milan, Italy)	1 tablet/day for 2 months	Significant increase in endothelial reactivity when compared with the control group

**Table 3:** Main results of the studies describing the effect of artichoke supplementation on blood pressure and body weight (BMI—body mass index; CG—control group; DBP—diastolic blood pressure; NAFLD—non-alcoholic fatty liver disease; NASH—non-alcoholic steatohepatitis; SBP—systolic blood pressure; TG—treatment group; y.o.—years old).

Authors (Year)	Population	Artichoke Product	Intervention	Main Results
Cicero et al. (2019a) [35]	40 subjects with pre-hypertension and dyslipidemia (TG: N = 20, 54 y.o.; CG: N = 20, 52 y.o.)	Dry extract of artichoke and Indian barberry (undisclosed composition)	1 tablet/day for 2 months	No significant change in SBP, DBP or BMI in either group or between groups
Cicero et al. (2019b) [108]	90 subjects with pre-hypertension and overweight (TG low dose: N = 30, 43 y.o.; TG high dose: N = 30, 45 y.o.; CG: N = 30, 44 y.o.)	Nutraceutical containing standardized bergamot extract [120 mg flavonoids), artichoke extract (2 mg 5-O-caffeoylquinic acid), 120 mg phytosterols and 20 mg vitamin C]	1 tablet/day (low dose) or 2 tablets/day (high dose) for 6 months	No significant change in SBP, DBP or BMI in either group or between groups
Roghani-Dehkordi & Kamkhah (2009) [104]	107 male subjects with stage 1 hypertension (TG 50 mg: N = 44 y.o.; TG 100 mg: N = 35, 44 y.o.; CG: N = 33, 44 y.o.)	Extract of concentrated artichoke leaf juice (50 mg or 100 mg)	2 tablets/day for 3 months	Significant reduction in SBP and DBP when compared with the control group. No significant difference in BMI when compared with the control group.
Ardalani et al. (2018) [76]	40 subjects with stage 1–2 hypertension and overweight/obesity medicated with captopril (TG: N = 20, 58 y.o.; CG: N = 20, 56 y.o.)	Artichoke leaf extract (500 mg)	2 capsules/day for 2 months	Significant reduction in SBP in both treatment and control groups with no significant differences between groups. Significant reduction in BMI in the treatment group when compared with the control group.
Fogacci et al. (2022) [107]	56 subjects (TG: N = 28, 54 y.o.; CG: N = 28, 54 y.o.)	Nutraceutical containing a bergamot extract (1000 mg), two artichoke extracts (120 mg), coenzyme Q10 (5 mg) and zinc (5 mg) (Eufortyn^®^ Colesterolo Plus, Milan, Italy)	1 tablet/day for 2 months	No significant change in SBP, DBP or BMI between treatment and control groups. Significant increase in endothelial reactivity index when compared to the control group. Significant reduction in waist circumference when compared to the control group.
Ferro et al. (2020) [103]	86 subjects with NAFLD (TG: N = 45, 53 y.o.; CG: N = 41, 51 y.o.)	Nutraceutical (300 mg) containing bergamot polyphenolic fraction, wild thistle extract, PUFA, bergamot pulp and albedo derivative	1 capsule /day for 3 months	No significant change in SBP or DBP in either group or between groups. Significant reduction in body weight and BMI in the treatment and control groups. Significant reduction when compared with the control group.
Panahi et al. (2018) [34]	89 subjects with NAFLD (TG: N = 49, 45.2 y.o.; CG: N = 40, 47.2 y.o.)	Artichoke leaf extract (200 mg; Cynarol^®^, Brussels, Belgium, standardized to contain 2 mg cynarin)	3 tablets/day for 2 months	Significant increase in SBP when compared with the control group. Significant decrease in BMI when compared with the control group.
Maurotti et al. (2024) [99]	32 subjects with non-alcoholic liver steatosis (TG: N = 16, 51 y.o.; CG: N = 16, 52 y.o.)	Supplement (300 mg) containing artichoke extract and bergamot polyphenol fraction	1 capsule/day for 3 months	No significant change in SBP, DBP or BMI between groups.
Rangboo et al. (2016) [105]	60 subjects with NASH (TG: N = 30, 47 y.o.; CG: N = 30, 49 y.o.)	Artichoke leaf extract	2700 mg/day (6 tablets) for 2 months	Significant reduction in SBP and body weight in treatment and control groups. No significant reduction in DBP in either group. No significant change in SBP or body weight between groups.

## Data Availability

Not applicable.
